# Further Views and Alternatives

**Published:** 1920-03-20

**Authors:** 


### CONTRIBUTIONS FROM PATIENTS.

### Further Views and Alternatives.

We have the pleasure to publish the following views from hospital chairmen and others, which have reached us since last week. Like those published in our issue of March 13, p. 572, they cannot fail to prove of great interest and to carry weight, coming as they do from the responsible authorities of hospitals of different types and situated in districts widely varying as to their population.

**Mr. R. J. Coles, Secretary, King's College Hospital, S.E.5.**

I am desired by the Chairman of King's College Hospital to say that the Committee of Management has not at present expressed any definite opinion as to the policy of asking each in-patient to contribute a definite sum per week. Since January of this year the enclosed form [stating the patient is prepared to contribute a sum (to be specified) per week towards cost of maintenance] has been forwarded with every admission order, and so far the results tend to show that 10s. is a good deal less than some patients can afford, while in other cases it is too high. The average amount promised by two-thirds of the patients admitted is 12s. 6d. per week, while the remaining one-third have promised nothing. The experiment has only been working about two months, and the Committee has not yet come to any definite conclusion. The principle of a general contribution by out-patients was adopted and came into force last October. Each out-patient contributes 6d., and 6d. extra for special treatment, such as massage, *x*-ray, or electrical; very few avail themselves of their right to appeal to the Almoner for exemption.

**Mr. E. G. Thorne, Vice-Chairman, Bolingbroke Hospital, Wandsworth Common, S.W.**

I am of opinion that it is not in the interest of the hospitals, as a rule, for patients to be asked to make any fixed contribution, as in that case the patients might consider that they are paying for the attention and care they get, and will not remember that, although they make a contribution, it is much below the expense to which the hospital is put for their maintenance. In the Bolingbroke Hospital the patients in the free wards are requested to make a donation according to their means, and of late we have found that they have responded to such an invitation to assist the hospital, and fully recognise that any such donation is not in any way commensurate with the expense to which the hospital is put through their being in-patients therein. I am not in favour of there being any fixed amount which an in-patient be expected to contribute.

**Mr. E. M Sneyd Kynnersley, Chairman, Royal Infirmary, Chester.**

We do not consider it possible to require any contribution from our in-patients. The resident medical staff and the secretary ascertain, as far as possible, whether patients are able to make any voluntary payment, and a fairly satisfactory sum is received. The question of admitting "paying patients" on a larger scale—with careful reservation of ample space for the poor—still occupies our attention.

Our present system is to encourage periodical contributions from workshops, etc., and the growth of this plan during the past year has been remarkable. This is, I think, partly due to the fact that the Governors have given considerably increased representation on the Board to the Saturday Hospital Committee, through whom the contributions are received, and that the working class are more directly interested in the institution. This is our alternative scheme, and it is general in the North of England. But, as you will see, this plan makes it impossible for us to levy any charge on our in-patients. They would reply, whether truly or not we could hardly ascertain, that they or their breadwinners are already contributing. A suggestion has been made that we should charge a registration fee of 6d. on each out-patient, as, we are told, is done at University College Hospital; but this would seem to necessitate the appointment of a clerk at £150 a year, and the balance, *if any*, would not compensate us for the ill-will produced.

**Mr. H. D. Stephenson, Chairman, Gravesend Hospital, Kent.**

I have been most interested in the proposal of the London Hospital to make a charge—for it is not a contribution—of 10s. per week for all patients, but it does appear to me to be jeopardising the voluntary principle of the hospitals.

Whilst admitting that the financial position of this hospital is not in a flourishing condition, the Committee are working very hard to keep things going, and are confident of success. A great change in the status of patients can be seen, for, in addition to treating the working man, the hospitals now get a fair proportion of the professional man—so-called the "middle class" in the provinces. My Committee have fully discussed the question of treating these patients, and found that when they were admitted to the general ward at no stipulated charge, whilst in hospital were full of good resolutions to assist financially, but such contributions were not always in proportion to the patients' known financial standing. The Committee decided upon an experiment. Three beds in the male and three in the female ward were screened off to form cubicles, and, in view of the cost per patient being approximately 52s. 3d. per week, fixed the charge at three guineas per week, it being left to the doctors' discretion as to who shall pay this sum. (These beds are used for general purposes when necessary.) This also gives the operating surgeon an opportunity of charging a fee, for, whilst reducing the cost to the patients by cutting out the high fees of a nursing home, it was deemed unfair that by admission to a general hospital surgeons should forgo fees; but when a patient cannot afford a cubicle and is admitted as an ordinary patient, no charge is made by the doctor. As to the ordinary patient, an appeal is, of course, made on the voluntary principle, and proves more or less successful; exemptions are made for the employees who subscribe to the Pay Day Fund, and their dependants. A special effort is being made to extend this fund on a much larger scale.

**Sir Harold Pink, Chairman of the Committee of Management, Royal Portsmouth Hospital.**

The only method to fill up the hospital coffers, it seems to me, is—give full representation on the Management Committee to the artisan classes, and expect them, in return, to assist the finances by inducing every worker to give not less than 1d. per week to the hospital. The

**Contributions from Patients**—(*continued*).

Portsmouth Royal Hospital has reorganised its constitution on these lines, and from present appearances there is every hope, by these small universal subscriptions in the district, that all expenses will be fully met. Patients are asked voluntarily to contribute something towards their maintenance, and the hospital receives on an average 5s. per patient, or about 1s. 8d. per week each. Great care, however, has to be taken in asking for this assistance; otherwise, so many people's sympathies will be alienated. The idea of absolutely *free* hospital attention is ingrained in the masses, but careful handling shows them that if a householder saves weekly by his relative being in hospital, this sum should be paid to the hospital funds. In many cases, it is found, there is no saving. If a husband—his income ceases. If a wife—the husband has to get help while the wife is away. Consequently, any charge should be very carefully handled, and only insisted on when the relative clearly benefits by the residence in hospital of the patient.

**Colonel W. Thornton, Chairman, Royal Berkshire Hospital, Reading.**

I have delayed answering your letter of March 4 until now, because I wished to consult some of my colleagues on the Board of the Royal Berkshire Hospital before expressing any views on the subject of levying a charge on in-patients at the hospitals in general and ours in particular.

(*a*) Speaking generally, I think the scheme of the London Hospital is the only one which will enable hospitals to carry on their beneficent work without calling on State aid. State aid means State control, and that, in my humble opinion, means loss of efficiency.

(*b*) As regards the Royal Berkshire Hospital, I may say that we have tapped every conceivable source of income we could think of, and the charitable public has responded nobly to our frequent appeals; but though we have considerably increased both the number of our subscribers and the amount subscribed, our expenditure in 1919 far exceeded our income. In these circumstances I can see nothing for it but to adopt the scheme of the London Hospital or to restrict our activities.

Of course there are objections to making a levy on in-patients, but it seems to me that the difficulties are not serious. It is said subscriptions may fall off, and the honorary medical staff may not look upon the arrangement with favour. But when once both subscribers and doctors realise that, failing something of the sort, the activities of the hospital must be greatly curtailed, I think that they will accept the inevitable.

The whole question has been relegated to a special committee, which in the near future will meet to consider the points involved and report.

**Col. H. d'Arch Breton, Chairman, St. Bartholomew's Hospital, Rochester.**

The present scheme of this hospital does not provide for admission by payment; but a new scheme now under consideration takes the necessary powers without specifying the amount, and, on receiving the approval of the Charity Commissioners, the precise terms will be decided by the Committee, if it should then be settled to exercise the powers. It will be understood that if any payment is taken for treatment the honorary medical staff will require a proportion to be allocated by the Medical Committee. As regards patients sent by the Schools Medical Officer a charge is made, of which the hospital takes 3s. 6d., that being the value of a subscriber's letter, and the balance goes to the Medical Committee. Our chief efforts at present are being directed to getting the industrial workers of the districts served to subscribe one penny a week. We are inclined to favour a charge for maintenance, and possibly for treatment, notwithstanding the practical difficulties in the way, and welcome the idea of making a small charge for out-patient attendances, if only because it will tend to discourage that class of applicant who have no real need of free treatment.

**Mr. S. C. Fryers, Secretary, The Royal Infirmary, Sunderland.**

The scheme adopted by the London Hospital is one which I think likely to find greater favour among the hospitals of the Metropolis than those of the provinces, where definite weekly contributions are paid direct to the infirmary by the workpeople. In my opinion the effect of such a charge as that suggested would be that these regular contributions would be, to a large extent, discontinued, and instead of contributing direct to the infirmary the workmen would subscribe to a fund of their own, out of which they would pay the 10s. demanded by the hospital for each of their cases admitted. Such a procedure would not, except in very few instances, operate to the advantage of the hospital funds. Were the scheme adopted at this institution the annual income from this source would not, at a liberal estimate, exceed about £4,500, whereas the amount derived from workpeople's subscriptions for 1919 was nearly £12,000, and this year it is anticipated that this will have increased to at least £15,000. In my opinion the better method is to bring home to employers and employees the need for contributions more commensurate with the present cost of upkeep and benefits received.

**Mr. T. B. Adams, Chairman, Wolverhampton and Staffordshire General Hospital.**

My opinion upon the decision of the London Hospital to ask each in-patient to contribute at least 10s. a week towards his maintenance is that, while I realise the financial position of the hospitals at the present time, I regret that it has not been found possible to devise some other method of raising revenue. My idea is that during sickness is the wrong time to ask for money from your patient. I believe in teaching the classes from whom hospital patients are drawn to adequately contribute to the hospitals regularly rather than calling upon them to do so during sickness.

**Capt. R. G. E. Whitney, Secretary, The National Hospital for Diseases of the Heart, Westmoreland Street, W.**

We have no fixed minimum of 10s. here. A payment of 5s. a week from a patient able and willing to pay this, but not more, has always been accepted. Patients, on the other hand, who can afford 15s. or £1 a week are asked to do so. So far as my experience goes of the working of such a system as this, I have nothing but praise for it, and I see no reason why it should not be generally adopted. There is no hardship to the patient provided the rate is adjusted to the position of the individual case; while if a proper plan of collection is put into force, there is no great increase of labour to the hospital, though the financial results well repay the trouble. I see no particular advantage in fixing a minimum of 10s. It is no more trouble to collect 7s. 6d. or 5s. than 10s., though the financial gain by accepting the smaller payments would be very considerable in so large a hospital as the London.

